# Giant colonic pseudo-diverticula importance of, and aids to radiological diagnosis: a case series

**DOI:** 10.1186/1757-1626-2-9314

**Published:** 2009-12-11

**Authors:** Godfrey T Chatora, Maruti Kumaran

**Affiliations:** 1 Department of Radiology, Nottingham University Hospitals NHS Trust, Queen's Medical Centre Campus, Derby Road Nottingham Nottinghamshire, NG7 2UH, UK

## Abstract

**Introduction:**

This case series illustrates the clinical presentation and radiological findings of two patients in whom the diagnosis of a Giant Colonic Diverticulum (GCD) was histologically confirmed. We also discuss the pathogenesis and differential diagnosis.

**Case presentation:**

In one case, the patient had no previous large bowel symptoms and intra operatively no other colonic diverticula were found. Both were treated with surgical resection.

**Conclusion:**

The preoperative diagnosis of GCD is made radiographically, and the importance of the early recognition of radiological findings, especially in asymptomatic disease, is emphasised.

## Background

A Giant Colonic Diverticulum (GCD) is a rare complication of colonic diverticulosis, a frequently occurring condition most often found in the sigmoid colon. This infrequent complication can easily be misinterpreted and is described only occasionally in the literature making it an interesting diagnostic problem both clinically and radiologically.

The preoperative diagnosis of GCD is made on plain abdominal radiography with findings of a large, smoothly marginated, round or oval, homogenous radiolucency in the abdomen that may contain an air-fluid level [1]. This may be in close apposition to the colon on barium enema examination. The Barium enema may document communication with the bowel lumen if patients are positioned properly to ensure uniform barium coating.

Importance of these findings and their early recognition is even greater in the absence of gastrointestinal complaints in view of the potential complications of perforation, obstruction or a volvulus, which occur in 10 to 19 per cent of cases [2]. In addition, giant diverticula carry a 2% risk of adenocarcinoma of the involved colon at the time of presentation.

The recommended treatment is excision of the diverticulum in continuity with the involved colonic segment.

## Case presentation

### Case 1

An 82-year-old Caucasian man was admitted with a 1-week history of worsening left upper quadrant pain. Apart from a 1-day history of constipation, he denied any previous bowel symptoms. His past medical history included sero-positive rheumatoid arthritis, chronic renal impairment secondary to gold injections, atrial fibrillation, chronic obstructive airways disease and previous right hip fracture.

On examination he was found to have a distended abdomen, with tenderness and resonant percussion note in the epigastrium and left upper quadrant. Blood results were unremarkable.

**Figure 1:**
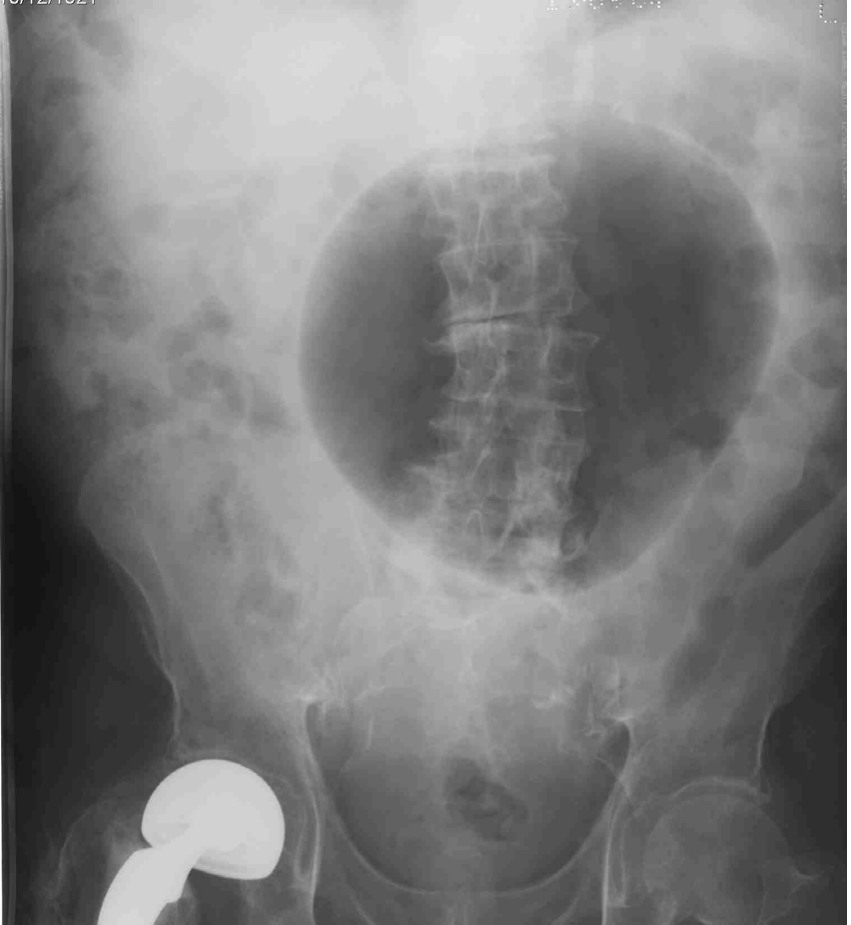
**Plain abdominal radiograph showing homogenous radiolucency that is smoothly marginated**.

**Figure 2:**
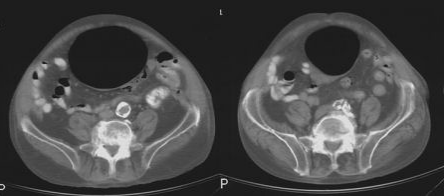
**Selected axial sections from CT scan, demonstrating a large gas filled viscus**.

An initial plain abdominal radiograph showed a large air filled cyst. [Figure 1: Plain abdominal radiograph showing homogenous radiolucency that is smoothly marginated] An intra-venous and oral contrast enhanced Computed tomography (CT) scan done on the same day demonstrated a large gas filled viscus. There was no mural enhancement or intraluminal contrast demonstrated. This was thought to represent a caecal volvulus or colonic diverticulum. [Figure 2: Selected axial sections from CT scan, demonstrating a large gas filled viscus]

Exploratory laparotomy was performed at which a large air filled cyst was found, adherent to sigmoid colon and bladder and containing only gas, no urine, fluid or faeces. No communication with the bowel or bladder lumen was demonstrated. The large bowel was otherwise free from diverticular disease. Excision of the cyst was performed.

Histology of the cyst showed muscularis mucosa and faecal material in granulation tissue within the lining of the cyst wall, making the cyst a type 1 giant colonic diverticulum or pseudodiverticulum [Table 1].

**Table 1:** cyst types

Type I	Pseudodiverticulum (pre-existing pulsion diverticulum)	Composed of granulation tissue and fibrous tissue, with chronic inflammatory cells and remnants of muscularis mucosa
Type II	Inflammatory diverticulum	Arises from local perforation and communicates with an abscess cavity. Wall is scar tissue only, no normal intestinal layers

Type III	True diverticulum	Contains all the layers of bowel wall

### Case 2

A 52-year-old Caucasian man with a longstanding history of Gastro-oesophageal reflux disease was under routine care in Gastroenterology clinic. He described a 5 week history of a mobile left upper abdominal mass associated with episodic pain and bloating worst after meals. He had experienced no weight loss and had regular bowel habit.

Over the next four months the patient developed worsening discomfort, alteration in bowel habit described as increased frequency of loose motions, and weight loss. Plain abdominal radiograph demonstrated a homogenous radiolucent oval structure in the left upper quadrant. Surgical referral and a contrast enhanced C.T scan were arranged.

**Figure 3:**
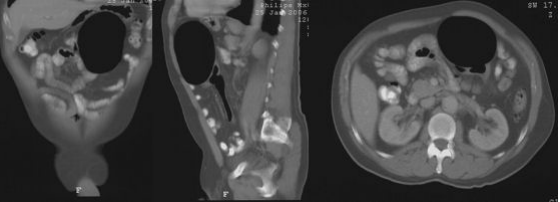
**Selected coronal, sagittal and axial sections from CT scan demonstrating a large gas filled structure that appeared to be arising from the top of the sigmoid colon**.

**Figure 4:**
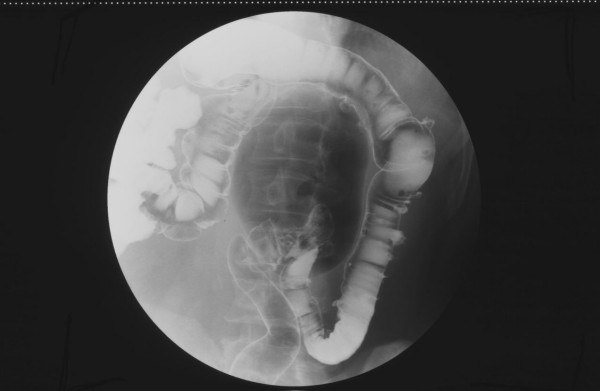
**Spot image from barium enema demonstrates an air collection immediately adjacent to the apex of the sigmoid loop and diverticular disease in the sigmoid and descending colon**.

The intra-venous and oral contrast enhanced CT scan demonstrated a large 12 cm gas filled structure in the left upper abdomen that appeared to be arising from the top of the sigmoid colon. There was no mural or intra-luminal enhancement, and this was associated with mild underlying wall thickening at the neck, and thought to represent a giant sigmoid diverticulum. [Figure 3: Selected coronal, sagittal and axial sections from CT scan demonstrating a large gas filled structure that appeared to be arising from the top of the sigmoid colon] A barium enema was requested and this showed that the air filled structure was immediately adjacent to the apex of the sigmoid loop but barium did not enter the cavity and it did not change size on air insufflation. [Figure 4: Spot image from barium enema demonstrates an air collection immediately adjacent to the apex of the sigmoid loop and diverticular disease in the sigmoid and descending colon] Diverticular disease was also noted in the sigmoid and descending colon.

Due to ongoing symptoms, the decision was made to perform an elective laparoscopy and sigmoid colectomy.

Pathologic examination demonstrated a cystic structure with a smooth lining, 11 cm in maximum diameter, with a wall thickness of 0.6-1 cm. There was no mucosa, and the wall was composed of dense fibrous tissue with foreign body reaction and acute and chronic inflammation. This made the cyst a type 1 giant colonic diverticulum or pseudodiverticulum. Diverticular disease was identified in the attached colon. The patient made an uneventful recovery.

## Discussion

### Epidemiology

The incidence of colonic diverticulosis is difficult to measure, as most patients remain asymptomatic. Studies show that up to 35 per cent of the population has colonic diverticulosis by age 65 [1]. Approximately 20 per cent become symptomatic, developing complications such as diverticulitis, haemorrhage, perforation, fistula formation, obstruction and giant cyst formation [3]. There is high geographic variability with the highest incidence in Western, industrialized nations, and the decreased intake of dietary fibre considered as a major etiologic factor.

Giant colonic diverticulum is a rare complication of this common disease with fewer than 150 cases reported in the literature. It affects men and women equally, occurs most commonly in patients 60-80 years of age, affecting the sigmoid colon in over 90% of cases [4].

### Pathophysiology

Hughes and Greene first reported this condition in 1953. Several theories have been proposed to explain its pathogenesis; the most widely accepted theory attributes the progressive dilatation to inflammation narrowing the diverticular ostium, creating a "ball-valve" mechanism, allowing air to enter the diverticulum, but not to return to the colonic lumen [4]. Other theories suggest that gas-producing micro organisms cause the diverticular expansion, or that a small colonic perforation causes pseudo cyst formation, followed by the "ball-valve" mechanism [5]. Pathologic examination is consistent, (see table 1)

### Clinical Presentation

Clinical presentation of GCD is very variable ranging from the asymptomatic patient, to patients presenting with an acute abdomen. Most patients however report abdominal pain, either acute or chronic. The other symptoms are less common and also non-specific, including fever, nausea, vomiting, constipation, diarrhoea, and haemorrhage [6]. Physical examination is similarly non-specific, revealing a soft, tympanic abdominal mass that may or may not be tender. Note is made of the example illustrated in Case 2 and figure 4, where the abnormality occurs in a redundant sigmoid colon and therefore is found in a location which is not classically thought of as being related to the sigmoid colon. Consequently, based on history and examination alone, the differential diagnosis includes many varying entities such as duodenal diverticulum, Meckel's diverticulum, duplication cyst, sigmoid volvulus, and emphysematous cholecystitis [7]. As the ostia are often are not directly visualized, endoscopy is not of benefit [8]. Ultimately its aetiology is uncertain and presentation is frequently non-specific, but the diagnosis is readily accomplished radiographically.

### Radiological Findings

Plain abdominal radiographs demonstrate a large, oval radiolucency, typically in the left lower quadrant, which may contain an air-fluid level in 25% of cases. A barium enema demonstrates the relation of the diverticulum to the bowel and results in the opacification of the diverticulum in 60% of patients [9]. When contrast flows into the diverticulum, irregular borders should raise the suspicion of chronic inflammatory or neoplastic changes.

The role of CT scanning has greatly increased and at present it is considered by many to be the diagnostic procedure of choice. On CT the GCD appears as a smooth-walled gas-containing structure ("balloon" sign) adjacent to the sigmoid colon [8]. The margin is thin and regular, and there is no contrast enhancement except in the presence of inflammation. The wall of the diverticulum can occasionally be thick whether from acute or chronic inflammation and could relate to pain.

Several MRI studies have demonstrated its efficacy in the diagnosis of diverticulitis and its associated complications including Giant Colonic Diverticula [10]. Heverhagen et al. reported that short time inversion recovery and true FISP (fast imaging with steady-state precession) sequences display pericolic exudation and oedema of the colic wall in diverticulitis. Case reports have shown how a thickened wall and air-fluid level in GCD was clearly observed on MRI. In addition, the infiltration into the surrounding fat and thickening of the adjacent mesentery were observed on MRI with fat suppression. MRI may however not be routinely required as CT is often sufficient to make the diagnosis and rule out complications.

Correct interpretation of radiological findings can exclude several important differentials. Duplication cysts are found in the lateral colon or on the posterior rectum but rarely on the sigmoid colon. A Meckel's diverticulum affects young children, in whom it is located in the distal ileum, and when complicated will specifically demonstrate the teardrop or blind-ending sac shape on ultrasound. When a mass with ring enhancement is observed in the female pelvic cavity, it is necessary to consider tubo-ovarian abscess, or a large uterine tumour, (which may contain gas if there is a fistula with the colonic lumen) as differential diagnoses. The most common appearance of tubo-ovarian abscess is that of a tubular, septate, cystic mass with uniform wall thickness and loss of fat planes between the mass and adjacent pelvic organs. On CT, a GCD is commonly gas-filled and oval whereas duplication cysts are usually fluid filled and fusiform.

Treatment ranges from conservative management to surgical intervention, because of the risk of complications surgical resection is felt by some to be the preferred management in all cases [5].

## Conclusion

The above illustrated cases demonstrate the efficacy of radiological findings in the diagnosis of Giant Colonic Diverticula in the appropriate clinical setting. The possibility should always be considered with such unusual radiological findings and rarity of the condition.

## Consent

Written informed consent was obtained from the patient for publication of this case report and accompanying images. A copy of the written consent is available for review by the Editor-in-Chief of this journal.

## Competing interests

The authors declare that they have no competing interests.

## Authors' contributions

GC: First/Main author, responsible for literature review. MK: Second author, responsible for reviewing and reporting imaging and supervising first author. Both authors read and approved the final manuscript.
